# Measured transverse‐axis dosimetric parameters of the model STM1251 I125 interstitial source

**DOI:** 10.1120/jacmp.v3i3.2564

**Published:** 2002-06-01

**Authors:** Z. Li, J. F. Williamson

**Affiliations:** ^1^ Department of Radiation Oncology, Mallinckrodt Institute of Radiology Washington University School of Medicine 660 S. Euclid Ave. St. Louis Missouri 63110

**Keywords:** permanent prostate implant, I125 seed source, TLD measurements, TG43 dosimetry formalism

## Abstract

The recent popularity of permanent implants for treatment of prostate cancer has created a large demand for low energy seed sources. Vendors have introduced new source designs to meet this demand. The AAPM has recommended that all low energy interstitial brachytherapy seed sources be subjected to independent dosimetric evaluations, preferably using experimental measurements as well as Monte Carlo calculations. This work presents the results of Thermo‐Luminescence Dosimeter (TLD) measurements of dosimetric parameters on the transverse axis of a new I125 seed source, the Source Tech Medical STM1251 I125 seed. Experimental measurements were performed in a Solid Water phantom, with the results corrected to values for liquid water using Monte Carlo calculated correction factors. The parameters measured include the dose rate constant and values of the radial dose function at distances of 0.5 cm through 5 cm. The measured dose rate constant in liquid water for the STM1251 I125 seed was 1.039 cGy/U‐hr. Measured radial dose function values agreed with Monte Carlo calculated ones to within 10%. These measurements therefore confirm the modeling and simulation of Monte Carlo calculations for this I125 source design, within the statistical uncertainties of the calculation and measurement techniques.

PACS number(s): 87.53.–j, 87.66.–a

## INTRODUCTION

As permanent implantation of low risk prostate cancer using I125 or Pd103 seed sources becomes a more widely adopted treatment option, new designs of I125 or Pd103 seeds have been introduced by many manufacturers. The American Association of Physicists in Medicine (AAPM)^1^ recommended that all such new source designs have their dosimetric parameters verified through independent dosimetric evaluations, using Monte Carlo calculations and/or experimental measurements. This report presents experimentally measured values of the dose rate constant in water and the radial dose function for the model STM1251 I125 seed, manufactured by Source Tech Medical, LLC, Illinois.

The measurement of brachytherapy source dose distributions is difficult because of the strict source/detector positioning accuracy requirement. Various detectors have been used for such purposes, including diode detectors,^2‐11^ diamond detector,^12^ and thermoluminescent dosimetry (TLD) chips and rods.^13^ ^–^ ^17^ Nonetheless, TLD measurements, using approximately water‐equivalent plastic phantoms, have become the de facto standard technique for this purpose.^18‐22^

In this work, we use standard TLD chips in a custom‐fabricated Solid Water^TM^ phantom to measure dose rate constant and radial dose function values of the STM1251 I125 source. The sources used in the measurements, phantom design, measurement techniques, and measurement results are presented in the subsequent sections of this paper.

## MATERIALS AND METHODS

### A. Materials and instruments

Three high‐activity STM1251 sources were used in this study. These sources have strengths of approximately 20 U (15.75 mCi), calibrated according to the revised NIST1999 standard by the University of Wisconsin ADCL. The source design has nominal external dimensions of 0.8 mm diameter and 4.5 mm length, with radioactive I125 deposited on a cylinder containing a gold marker in the center. A detailed description of the source construction is presented in the work of Kirov and Williamson.^23^

The measurements performed in this study use LiF TLD chips of $3\times 3\times 1{\text{mm}}^{3}$ and $1\times 1\times 1{\text{mm}}^{3}$, acquired from Harshaw/Bicron Radiation Measurement Products, Dayton, OH. The smaller chips were placed at 0.5 to 2 cm radial distances away from the center of the I125 source, while the large chips are placed at further distances, such that volume‐averaging effects of the dosimeters may be minimized. An automatic oven (Thermodyne model F47900, Barnstead/ Thermodyne, Dubuque, IA), able to automatically control the oven temperature for preset length of time, was used for the annealing of the TLD chips. The TLD chips were read using a Bicron 5000 automatic TLD reader, which automatically reads up to 50 TLD chips.

A plastic phantom, machined from Solid Water^TM^‐material (Radiation Measurements, Inc., Madison, WI), was used for the TLD measurements. The phantom has four spiral patterns of holes for placing TLD chips. The holes are sized to fit $1\times 1\times 1{\text{mm}}^{3}$ TLD chips at distances up to 2 cm away from the source, and $3\times 3\times 3{\text{mm}}^{3}$ TLD chips at larger distances. Individual plugs are available to cover up the holes not filled with TLD chips during experimental measurements. A cylindrical source holder fits snugly into a hole in the center of the spiral pattern, and is used to place the source on its end accurately in the center of the spiral patterns. The source holder was machined from the same solid water material as the phantom. It helps eliminate potential damages to the seed, and allows more accurate timing of seed insertion and irradiation, since the entire seed holder can be removed and placed in the solid water phantom quickly. During the measurement experiments the solid water phantom was further surrounded by additional solid water pieces to provide adequate scattering medium for the I125 photons.

### B. Measurement Procedure

The TLD chip annealing and reading procedure reported by Meigooni *et al*.^24^ was used in this work. Briefly, this procedure calls for annealing the chips at 400 °C for 1 h, followed by 80 ° C for 24 h pre‐irradiation annealing, with rapid cooling of the chips in between. No post‐irradiation annealing was performed, though all chips were read at 24 h post‐irradiation The automatic TLD reader was connected to a nitrogen tank so that the TLD planchet is drenched by nitrogen during reading.

Individual TLD chip response correction factors were obtained by irradiating the TLD chips in a solid water phantom under a flat external beam radiation field of 6 MV *x*‐rays. The correction factors were then applied to the readings of individual TLD chips following their irradiation by the I125 sources. Calibration factors for the TLD chips were obtained by irradiating TLD chips at ranges between 5 and 250 cGy, in a 6 MV x‐ray field. The readings of the calibration TLD chips were converted to calibration factors for I125 energy using a multiplicative conversion factor of 1.41, as reported by Meigooni *et al*.^25^ These factors constitute a nonlinear correction function that is used to convert readings of the I125‐irradiated TLD chips to dose. Depending on the distances of the TLD chips away from the source, the TLD chips were exposed to different lengths of times to the source, so as to maintain the maximum doses to less than 250 cGy.

The readings of TLD chips, irradiated by the I125 seeds, are converted to dose rates in solid water by correction for source decay during irradiation. The complete dose rate calculation for a TLD chip, placed at a radial distance of *r* cm from center of the I125 source, is then given by the following:

(1) $$\dot{D}(r)=\frac{CF_{chip}\cdot Rdg_{125_{I}}(r)}{1.41\cdot (Rdg_{6x}/cGy)\cdot T_{eff}\cdot (1-e^{0.693\cdot t/59.6})},$$

where ${\text{CF}}_{\text{chip}}$ is the chip correction factor; *Rdg* I125(*r*) is the TLD reading, corrected for background, of the chip irradiated in I125 radiation field, at distance *r* from center of the source; ${\text{Rdg}}_{6x}/cGy$ is the TLD chip calibration factor in 6 MV x‐ray beam; $T_{\text{eff}}$ is the effective half life of I125 source in hours; and *t* is the length of irradiation time in days for the TLD chip.

The measured dose rates per unit source strength in solid water are further obtained by dividing the dose rates by the strength of the source at beginning of irradiation experiment for each chip. These values are then converted to dose rates in liquid water using solid water to liquid water conversion factors calculated using the same Monte Carlo code package, cross section data, and geometric model for the seed as in those in Kirov and Williamson.^23^ The vendor‐specified chemical composition (H:C:N:O:C1:Ca=8.1%:67.2%:2.4%:19.8%:0.13%:2.3% by mass) of the solid water material was used in these Monte Carlo calculations. Dose rate constant of the source, as well as radial dose function values for the source at 0.5 to 5 cm from center of the source, are further calculated from these dose rate values, as defined in the AAPM Task Group 43 report.^26^ The dose rate constant in liquid water of the STM1251 source was calculated by applying a Monte Carlo calculated multiplicative correction factor of 1.034 to the measured dose rate constant in solid water for the source.

## RESULTS AND DISCUSSION

### A. Dose rate constant in water

The measurements yielded a dose rate constant in liquid water for the STM1251 source of 1.039 cGy/U‐hr, averaged over nine measurement data points. The standard deviation of the measured data was 2.5%. As discussed by Nath and Yue,^21^ and Meigooni *et al*.,^19^ TLD experiment results are plagued by measurement uncertainties introduced from source strength calibration at approximately 1%, LiF TLD energy response with 5% uncertainties, solid water to liquid water conversion with 3% uncertainties, TLD chip and seed positioning uncertainty of 1%, as well as TLD reading to dose response calibration uncertainties of about 3%. Combining these sources of errors in quadrature, we estimate the uncertainties associated with our TLD measurement of the dose rate constant in water for the STM1251 I125 source to be approximately 7%, not including uncertainties associated with the chemical composition of solid water phantom material and its impact on conversion of dose in solid water to dose in liquid water. As discussed by Patel *et al*.,^27^ experimentally determined calcium content of solid water material may differ from the specified value by as much as 30%, causing additional uncertainties of up to 5% at 1 cm and 25% at 5 cm on the calculated solid‐to‐liquid‐water conversion factors. While our measured dose rate constant of 1.039 cGy/U‐hr for this source differs by approximately 6% from the Monte Carlo calculated value^23^ of 0.98 cGy/U‐hr, the difference falls within one standard deviation of the TLD measurement results.

### B. Radial dose function values

As described in the AAPM TG43 report,^26^ radial dose function values are calculated from the dose rates at points along its transverse axis, and the geometric function values of the source. The model STM1251 source has an active source length of 0.38 cm, which is used in the calculation of geometric function values for this source using the unfiltered line source equation.^26^ The calculated geometry function values are tabulated in Table I. It should be noted that these geometry function values are specific to this source design, and should be used for inputting into computerized treatment planning system as part of source data for the STM1251 source.

**Table I:** Geometric function values for the STM1251 I125 source, with a 0.38 cm active source length.

Radial distance *r* (cm)	0.5	1.0	1.5	2.0	3.0	3.0	5.0
	0.956	0.988	0.995	0.997	0.999	0.999	1.000

Our measurements of the radial dose function values for the STM1251 source were performed for radial distances up to 5 cm away from the source. Since the measurements were performed in solid water phantom, a set of distance‐dependent solid water to liquid water conversion factors was calculated using Monte Carlo simulations (as described previously) as ratios of Monte Carlo calculated dose to liquid water to calculated dose in solid water at a given point of interest, namely, $CF=D_{liquid\;water}(r)/{\dot{D}}_{solid\;water}(r)\;$. These conversion factors are then used to obtain radial dose function values in liquid water for the STM source. These conversion factors are listed in Table II.

**Table II:** Solid water to liquid water multiplicative conversion factors.

Radial distance *r* (cm)	0.5	1.0	1.5	2.0	3.0	3.0	5.0
Conversion factor	1.015	1.034	1.053	1.076	1.114	1.144	1.176

With these correction factors applied, the measured radial dose function values for the STM1251 source, averaged over six or more measurement data points, are summarized in Table III, together with the Monte Carlo calculated radial dose function values for the same source, and those for the model 6711 and model 6702 sources. The measurement standard deviation and (maximum) Monte Carlo calculation uncertainties are listed in the same table. The Monte Carlo calculation uncertainties, as discussed by Kirov and Williamson,^23^ include primarily uncertainties in the photon interaction cross‐section data.

**Table III:** Radial dose function values of the STM1251 source.

Distance (cm)	STM1251/measured *g*(*r*)	Standard deviation%	STM1251/calculated *g*(*r*)23	Uncertainty^23^%	Model 6711 *g*(*r*)^26^	Model 6702 *g*(*r*)^26^
0.5	1.033	3.44	1.033	5.0	1.040	1.040
1.0	1.000		1.000		1.000	1.000
1.5	0.929	6.53	0.937	5.0	0.926	0.934
2.0	0.905	3.90	0.856	5.0	0.832	0.851
3.0	0.748	6.53	0.691	5.0	0.632	0.670
4.0	0.613	3.47	0.540	5.0	0.463	0.511
5.0	0.466	10.00	0.415	5.0	0.344	0.389

The measured radial dose function values carry significantly large uncertainties as seen in the measurement standard deviations. At the same time, the measured values indicate the same trend of the radial dose function values of the STM1251 source as that shown by the Monte Carlo calculations, relative to the radial dose function values of model 6711 and model 6702 sources. That is, the STM1251 source appears to be slightly more “penetrating,” with larger *g(r)* values than those of the other two sources. Since the model 6711 source contains a silver marker, and hence has a spectrum with lower average energy than the model 6702 and STM1251 sources due to the low energy silver x‐ray photons, it is expected that the STM1251 source would have dosimetric properties similar to those of the model 6702 source. The measured radial dose function values support this observation. Since our solid‐to‐liquid water corrections were based upon the nominal Solid Water composition provided by the vendor, the discrepancy between measurement and simulation may be explained, in part, by uncertainties in the calcium content of Solid Water. Patel *et al*.^27^ demonstrated that his Solid Water material contained significantly less calcium than specified by the vendor. If so, the true solid‐to‐liquid water corrections may be smaller than those listed in Table II, which would make the measured *g*(*r*) function less penetrating.

## CONCLUSIONS

TLD measurements were carried out for the determination of dosimetric parameters of a different I125 source, the STM1251 seed source, along its transverse axis. The measurements serve to validate the modeling and calculations of previous Monte Carlo simulations for the same source within the statistical uncertainties of the calculation and measurement techniques. Specifically, the agreement between the measured and calculated dose rate constants for the STM1251 source was consistent with the uncertainties of the calculated and measured parameters.

## ACKNOWLEDGMENT

This work was supported, in part, by a grant from Source Tech Medical, LLC.
